# Near-Infrared Fluorescence Probes for Fluorescence-Guided Surgery: Current Clinical Probes and Emerging Candidates

**DOI:** 10.3390/molecules31193466

**Published:** 2026-09-29

**Authors:** Zhuoying Kang, Xujing Jiang, Guangxuan Jin, Jue Jin, Yuding Zhang, Ziying Wei, Guojun Xiong, Dan Wu

**Affiliations:** 1 International College of Pharmaceutical Innovation, Soochow University, Suzhou 215123, China; 20244259009@stu.suda.edu.cn (Z.K.); 2453401013@stu.suda.edu.cn (X.J.); 2453401051@stu.suda.edu.cn (G.J.); 2453401022@stu.suda.edu.cn (J.J.); 20254026006@stu.suda.edu.cn (Y.Z.); wzy1218@outlook.com (Z.W.); gjxiong@suda.edu.cn (G.X.); 2 School of Pharmacy and Biomolecular Sciences, Royal College of Surgeons in Ireland, 123 St Stephen’s Green, D02 YN77 Dublin, Ireland; 3 Department of Chemistry, Royal College of Surgeons in Ireland, 123 St Stephen’s Green, D02 YN77 Dublin, Ireland

**Keywords:** fluorescence-guided surgery, near-infrared imaging, molecular probes, clinical translation

## Abstract

Fluorescence-guided surgery (FGS) enables real-time visualization of tumor margins and microscopic residual lesions, thereby improving the completeness and precision of tumor resection. Paralleling these clinical advancements, fluorescence probes have shown significant progress in intraoperative imaging applications. Several probes approved by the U.S. Food and Drug Administration (FDA), including indocyanine green (ICG), 5-aminolevulinic acid (5-ALA), sodium fluorescein, methylene blue (MB), pafolacianine (OTL-38), and pegulicianine (LUM015), have been widely used in various solid tumor surgeries, while Akalux received approval in Japan in 2020 for near-infrared photoimmunotherapy. Beyond these established agents, a large number of novel probes are currently undergoing clinical evaluation, with design trends shifting toward active targeting and microenvironment responsiveness. This review provides an overview of FDA-approved probes and systematically summarizes representative probes in phase I-III clinical trials, focusing on their imaging mechanisms, clinical evidence, and translational challenges.

## 1. Introduction

In the 21st century, cancer remains a major global public health challenge [1]. Over recent decades, a variety of therapeutic strategies have been developed and applied in clinical oncology [2,3]. Despite significant advances in cancer treatment, surgical resection continues to serve as the cornerstone for curative treatment of most solid tumors [4]. The primary goal of oncologic surgery is to achieve complete removal of malignant tissues while preserving as much normal tissue as possible. However, for tumors with extensive metastasis or those located in anatomically complex regions, residual cancer cells often remain after surgery, leading to positive surgical margins [5,6]. Positive margins are strongly associated with an increased risk of local recurrence and poor prognosis, highlighting a critical unmet need for enhanced intraoperative guidance. Intraoperative tumor delineation has relied heavily on the surgeon’s tactile feedback, visual inspection, and preoperative imaging modalities such as Computed Tomography (CT), Magnetic Resonance Imaging (MRI), and Positron Emission Tomography (PET) (Figure 1). While indispensable for preoperative planning, these technologies are hindered by bulky instrumentation, high costs, and a lack of real-time intraoperative utility, and it is difficult to identify tumor boundaries from surrounding normal tissues with the naked eye during the surgery process [7]. Similarly, adjuncts like selective frozen-section analysis are constrained by sampling bias and the risk of false-negative results [8], while intraoperative ultrasound requires specific anatomical conditions that are not universally applicable [9]. Fluorescence imaging has emerged as a valuable adjunct for real-time visualization and delineation of solid tumors during surgical resection (Figure 1). Compared with conventional imaging modalities, fluorescence imaging offers several advantages, including high spatial resolution, real-time image acquisition, simple and compact instrumentation, and the potential for repeated imaging throughout the surgical procedure. These characteristics make fluorescence imaging particularly attractive for intraoperative tumor guidance. Intraoperative fluorescence imaging enables the visualization of small lesions and precise delineation of tumor margins, thereby facilitating more complete tumor removal. Fluorescent probes can generally be classified according to their emission wavelengths into visible-light, near-infrared-I (NIR-I, approximately 700–900 nm), and near-infrared-II (NIR-II, approximately 900–1700 nm) regions. Compared with visible-light fluorescence, NIR fluorescence exhibits reduced tissue autofluorescence and light scattering, and offers higher sensitivity, thereby improving the signal-to-background ratio and tissue penetration [10,11].

This review focuses on molecular probes used in FGS. We first summarize the pharmacokinetic profiles and clinical applications of established FDA-approved probes [12], then provide an overview of probes currently under clinical investigation. Finally, we discuss the future prospects of fluorescent probes in FGS applications.

## 2. FDA Approved Probes

The current clinical paradigm of FGS is underpinned by a cohort of FDA-approved fluorescent agents. Owing to their well-established safety profiles and extensive clinical experience, these agents have become fundamental tools in FGS. In the following sections, the molecular characteristics including chemical structure, photophysical properties (Figure 2), and the clinical evidence of each probe will be systematically described.

### 2.1. Nonspecific Probes

#### 2.1.1. Indocyanine Green

ICG is a classical NIR fluorescent dye. Its chemical structure consists of two indolenine rings linked by a polymethine conjugated chain, with terminal sulfonate groups that confer good water solubility. It has an excitation wavelength within the range of 600–900 nm, with an emission wavelength within the range of 750–950 nm [13], which contribute to reduced tissue scattering and improved penetration for in vivo imaging. In terms of its pharmacokinetics, ICG is not absorbed through the gastrointestinal tract and is instead administered intravenously. Following injection, it rapidly binds to plasma proteins, then it is selectively taken up by hepatocytes and excreted into the bile, resulting in a rapid elimination with a plasma half-life of approximately 3–4 min [14,15].

ICG was approved by the FDA in 1959 [16] and initially used for vascular studies [17] before its applications were expanded to broader medical imaging and surgical diagnostics. Following its successful clinical application in vascular imaging, ICG-based NIR fluorescence imaging has been used in anatomical structure identification. Wang et al. investigated the clinical application of ICG in laparoscopic common bile duct exploration. The study demonstrated that preoperative intravenous ICG administration provided clear biliary delineation under near-infrared imaging [18]. Beyond anatomical guidance, ICG has been investigated for tumor localization and resection guidance. Predina et al. conducted a clinical trial evaluating ICG fluorescence imaging during anterior mediastinal tumor resection. The study demonstrated its good safety in anterior mediastinal tumors [19]. In clinical studies of early-stage cervical cancer, ICG also enabled clear intraoperative visualization of key pelvic nerves [20]. As clinical applications have expanded, fluorescence-guided surgery has evolved from intraoperative visualization and anatomical localization toward the identification of occult lesions and the optimization of surgical completeness. In lung cancer, ICG-mediated intraoperative imaging has been shown to identify pulmonary metastases that were not identified by conventional methods, thereby influencing postoperative treatment strategies [21]. Building on its ability to reveal occult disease, the clinical utility of ICG fluorescence imaging has further advanced toward real-time assessment of surgical margins, an essential component of achieving complete tumor resection. A multicenter prospective cohort study conducted by Achterberg et al. demonstrated that the application of ICG fluorescence imaging in minimally invasive colorectal liver metastasis resection increased the R0 resection rate, indicating that ICG fluorescence imaging aids in the detection of additional occult lesions [22]. Although ICG-based imaging provides valuable intraoperative guidance, it lacks tumor specificity and relies on passive accumulation. This nonspecific nature, coupled with rapid clearance, leads to variable tumor-to-background ratios and narrow imaging windows. Because it tracks dye distribution rather than molecular activity, ICG struggles to reliably distinguish malignant from inflamed or normal tissues. To address this limitation, recent studies have explored strategies that combine active tumor targeting with the reduction in nonspecific interactions in vivo. Dou et al. developed an orthogonally engineered human serum albumin (HSA) platform for the targeted visualization and phototherapy of hepatocellular carcinoma [23]. The engineered albumin incorporated a glypican-3 (GPC-3)-targeting peptide to facilitate selective delivery to GPC-3-positive liver cancer cells, while an additional peptide was introduced to attenuate macrophage phagocytosis, thereby reducing nonspecific uptake by immune cells. This dual-functional design improved tumor targeting and enhanced tumor-to-background contrast, demonstrating that effective fluorescence-guided tumor imaging requires not only specific recognition of tumor-associated biomarkers but also control of nonspecific interactions within the tumor microenvironment.

#### 2.1.2. Sodium Fluorescein

Sodium fluorescein is a small-molecule, water-soluble fluorescent dye belonging to the xanthene derivatives [24]. It exhibits a maximum excitation wavelength at 465–490 nm, with a corresponding emission peak at 510–530 nm. In aqueous solution, it predominantly exists in an ionic form, and its molecular structure and fluorescence spectrum are influenced by the pH of the environment [25]. Due to its small size and high water solubility, sodium fluorescein rapidly distributes within the vascular system after intravenous administration. Its fluorescence signal is highly dependent on vascular permeability and barrier integrity, as leakage of fluorescein molecules from compromised vessels leads to increased extravascular fluorescence [26,27]. These properties have enabled its application in ophthalmic imaging.

Sodium fluorescein was approved by the FDA in 1976 for diagnostic fluorescein angiography or angioscopy of the retina and iris vasculature [28,29]. Beyond retinal vascular imaging, its applications have expanded to other ophthalmic evaluations, including assessment of corneal epithelial damage and ocular surface disorders [30]. With its ability to highlight tissue abnormalities and leakage sites, the clinical application of sodium fluorescein has extended beyond ophthalmology, particularly in neurosurgical procedures. Minkin et al. investigated its intraoperative utility in benign neuroepithelial brain tumors, demonstrating its potential for tumor visualization during surgery [31]. It can also serve as a tracer to enhance visualization of central nervous system tumors, including recurrent glioblastoma and pediatric posterior fossa tumors [32,33,34]. In addition to tumor visualization, sodium fluorescein can also be administered intrathecally for intraoperative localization of cerebrospinal fluid (CSF) leaks [35,36]. However, its intrathecal use in neurosurgical procedures remains an off-label application. Compared with conventional imaging modalities, its advantage in overall leak detection rates remains limited [37]. Overall, with careful evaluation of patient allergy history and strict adherence to standardized protocols, it represents a feasible option for intraoperative fluorescence guidance [38].

#### 2.1.3. Methylene Blue

MB is a thiazine-based cationic dye. In 2011, MB was evaluated by the European Medicines Agency and subsequently authorized by the European Commission [39]. In 2016, its injectable formulation was approved by the FDA [40] for acquired methemoglobinemia, whereas its use as a tumor tracer remains off-label and largely confined to experimental studies. Varghese et al. demonstrated that MB achieves a high detection rate of sentinel lymph nodes without inducing severe allergic reactions [41]. Similarly, MB-assisted SLN mapping provided stable and visually distinguishable lymph node staining in patients with early-stage cervical cancer [42]. Beyond SLN mapping, MB has been further applied for intraoperative tissue identification. In parathyroid adenoma, low-dose intravenous administration of MB combined with NIR fluorescence imaging can assist in intraoperative identification of parathyroid tissue and lesions, thereby supporting optimization of resection strategies [43,44]. Galema et al. further reported that MB-based NIR fluorescence imaging facilitated the identification and resection guidance of metastatic small intestinal neuroendocrine tumors [45]. These findings showed that MB-based NIR imaging is valuable for intraoperative localization of lymph nodes and selected lesions; however, its limited tumor specificity restricts its application for primary tumor detection.

### 2.2. Targeted Probes

#### 2.2.1. Pafolacianine (OTL-38)

Pafolacianine is a targeted molecular probe composed of a folate analog ligand conjugated to an NIR fluorescent dye. It exhibits favorable water solubility and excellent optical properties, with a peak excitation wavelength of 774 nm and an emission maximum at 794 nm [46]. Through folate receptor targeting, pafolacianine selectively binds to folate receptor alpha (FRα)-positive cells and accumulates intracellularly via receptor-mediated endocytosis. The clinical translation of pafolacianine has been primarily demonstrated in ovarian cancer. In 2021, pafolacianine was approved by the FDA for intraoperative identification of malignant lesions in patients with ovarian cancer [47].

A first-in-human translational study showed that pafolacianine could be safely administered and enabled real-time intraoperative visualization of FRα-positive ovarian cancer lesions [48]. Subsequent phase II and III trials further confirmed its favorable safety profile, high lesion detection efficiency, and clinical benefits, with mild drug-related adverse events reported [49,50]. Beyond ovarian cancer, the application of pafolacianine has been extended to other FRα-overexpressing tumors [51] including renal cell carcinoma [52], gastric adenocarcinoma [53], pituitary adenoma [54], and endometrial cancer [55], demonstrating its potential for lesion localization, surgical guidance, and improved clinical decision-making. Nevertheless, further large-scale trials and technical improvements are needed to address current limitations such as false positives associated with inflammations and to define its future integration into precision oncology workflows.

#### 2.2.2. Akalux

Akalux is an antibody–photosensitizer conjugate developed for near-infrared photoimmunotherapy (NIR-PIT) [56]. It consists of the epidermal growth factor receptor (EGFR)-targeting monoclonal antibody cetuximab conjugated with the photosensitizer IRDye^®^700DX, which exhibits an absorption peak at 689–690 nm and an emission peak at 700–702 nm, allowing activation by 690 nm NIR light to induce therapeutic effects [57,58]. Following intravenous administration, Akalux selectively binds to tumor cells overexpressing EGFR. Upon NIR irradiation, the activated photosensitizer induces rapid disruption of the cell membrane, resulting in selective necrosis of targeted tumor cells [59,60,61]. Akalux was approved in Japan in 2020 for the treatment of unresectable locally advanced or locally recurrent head and neck cancer [62].

In a single-center, open-label phase I study, Akalux-based photoimmunotherapy was administered to three patients with heavily pretreated recurrent head and neck squamous cell carcinoma (rHNSCC). The treatment demonstrated a manageable safety profile, and two of the three patients achieved partial responses, indicating its antitumor activity [59]. Cognetti et al. further evaluated Akalux in a multicenter, open-label phase I/IIa study in patients with locoregional rHNSCC, supporting its potential as a therapeutic option [63]. Beyond rHNSCC, the clinical development of Akalux is being extended to other solid tumors. For example, an ongoing clinical trial (NCT06943664) is evaluating the combination of Akalux-based photoimmunotherapy with anti-PD-1 therapy in patients with refractory metastatic stage IIIB-IV non-small cell lung cancer, aiming to assess its efficacy and safety [64]. Akalux represents a clinically translated NIR-triggered therapeutic platform that enables selective tumor cell ablation through antibody-mediated targeting and light-controlled activation.

### 2.3. Activatable Probes

#### 2.3.1. 5-Aminolevulinic Acid (5-ALA)

5-ALA is a non-proteinogenic small-molecule amino acid that functions as a metabolic precursor for fluorescent protoporphyrin IX (PpIX). 5-ALA itself is weakly fluorescent and its imaging signal is generated through intracellular metabolic conversion to protoporphyrin IX (PpIX) [65], which accumulates in tumor cells and enables intraoperative visualization. The clinical application of 5-ALA has been primarily established in fluorescence-guided resection of malignant gliomas. Stummer et al. demonstrated in a multicenter randomized phase III clinical trial that 5-ALA-assisted surgery improved the extent of malignant glioma resection [66]. Based on its clinical benefits, 5-ALA was approved within the European regulatory system in 2007 for fluorescence-guided resection of high-grade gliomas [67], followed by approval in Japan in 2013 [68] and by the FDA in 2017 as an adjunct for intraoperative optical imaging of high-grade gliomas [69].

Beyond central nervous system tumors, 5-ALA-mediated photodynamic diagnosis has been extended to other solid tumors. In bladder cancer, Yamamoto et al. demonstrated that 5-ALA-assisted fluorescence cystoscopy provided improved detection sensitivity for carcinoma in situ lesions with an acceptable safety profile [70]. Furthermore, Hexaminolevulinate (HAL), a derivative of 5-ALA, has been successfully applied in blue-light cystoscopy for bladder cancer detection [71], which shows the prodrug modification of 5-ALA has expanded its clinical applications. 5-ALA represents a metabolic activation strategy that converts tumor-associated metabolic alterations into fluorescence signals. However, its application remains largely limited to tumors with enhanced PpIX accumulation, and further optimization is required to improve tumor selectivity in broader clinical settings.

#### 2.3.2. LUM015 (Pegulicianine, LUMISIGHT)

Pegulicianine is a protease-activatable NIR fluorescent imaging probe that generates intraoperative signals through selective activation by tumor-associated proteases within the tumor microenvironment. Structurally, pegulicianine consists of a Cy5 fluorophore, a protease-cleavable peptide linker, a quencher (QSY21), and a polyethylene glycol (PEG) moiety. In its intact form, the Cy5 fluorescence is quenched. Upon proteolytic cleavage in tumor tissues, fluorescence is restored, enabling selective tumor visualization. The activated fluorescence signal exhibits an absorption maximum at 650 nm and an emission peak at 670–675 nm [72,73]. The clinical translation of pegulicianine has been primarily demonstrated in fluorescence-guided breast-conserving surgery. A phase I study conducted by Whitley et al. first established the feasibility of pegulicianine for intraoperative imaging of soft-tissue sarcoma and breast cancer models [74]. Following these early clinical investigations, pegulicianine was approved by the FDA in 2024 under the generic name LUMISIGHT for intraoperative identification of residual tumor tissue during breast-conserving surgery [72]. Subsequent clinical studies further confirmed its ability to provide real-time visualization of residual tumor tissue within the surgical cavity and improve intraoperative margin assessment, as demonstrated in a phase III multicenter randomized trial [75,76]. As one of the first clinically translated activatable fluorophores, pegulicianine enhances tumor specificity by converting tumor-associated protease activity into fluorescence signals through tumor microenvironment-responsive activation. However, its current clinical application remains largely limited to breast cancer surgery.

The clinical evidence of these probes is summarized in Table 1.

## 3. Probes in Clinical Trials

Beyond clinically approved fluorescent probes, a growing number of investigational agents are being evaluated in clinical trials to further improve the precision and versatility of fluorescence-guided surgery. Based on their clinical development stage, they can be categorized into phase I, II, and III clinical trials.

### 3.1. Probes in Phase I Clinical Trial

Primary objectives in phase I clinical trials focused on evaluating safety and preliminary pharmacokinetic characteristics. The molecular structures, excitation wavelengths, and emission wavelengths of representative probes in phase I studies are summarized in Figure 3.

#### 3.1.1. Antibody-Based Targeted Probes in Phase I Clinical Trial

Among phase I clinical candidates, antibody-based fluorescent probes represent one of the most extensively investigated strategies due to their high target specificity and clinical feasibility. These probes utilize monoclonal antibodies to recognize tumor-associated antigens and enable selective fluorescence-guided surgery.

Panitumumab-IRDye800 is an EGFR-targeted fluorescent probe generated by conjugating the anti-EGFR monoclonal antibody panitumumab with IRDye800CW at a substitution ratio of 1:1–1:2 [77]. This probe is primarily applied in FGS for EGFR-overexpressing tumors, including high-grade gliomas and head and neck squamous cell carcinoma (HNSCC) [77]. In a phase I clinical trial for high-grade gliomas, the probe demonstrated the ability to penetrate the disrupted blood–brain barrier and selectively label tumor cells, supporting its potential for intraoperative tumor visualization [78].

Bevacizumab-IRDye800CW is a vascular endothelial growth factor A (VEGF-A)-targeted NIR fluorescent tracer, formed by covalent conjugation of bevacizumab with IRDye800CW and exhibits excitation and emission wavelengths of 774 nm and 789 nm, respectively. It had shown limited imaging specificity in pancreatic cancer due to the difficulty distinguishing tumor tissue from normal or inflamed pancreatic tissue [79]. But a phase I trial (NCT02583568) in primary breast cancer showed the probe selectively accumulates in tumor tissue via VEGF-A targeting, with good safety and specificity for tumor imaging [80].

#### 3.1.2. Peptide-Based Targeted Probes in Phase I Clinical Trial

In addition to antibody-based approaches, smaller targeting ligands have been explored to improve tumor penetration and enable imaging of diverse molecular targets.

HS-196 is a small-molecule inhibitor–NIR fluorescent probe developed by conjugating a heat shock protein 90 (Hsp90) inhibitor to an NIR fluorophore via a linker. It selectively targets Hsp90, which is overexpressed in malignant tumors, enabling tumor-specific fluorescence imaging [81]. A phase I clinical trial (NCT03333031) has been initiated to evaluate its safety and feasibility for precise tumor navigation in breast cancer.

LS301 is a peptide-dye conjugate composed of the NIR fluorophore cypate and a cyclic peptide c(DCGRDSPC)K, with excitation and emission maxima at 780 nm and 830 nm, respectively [82]. It selectively binds to phosphorylated Annexin A2 (pANXA2) on the cell surface and is internalized via lipid raft-associated, clathrin-dependent endocytosis, resulting in accumulation in tumors [83,84]. Following promising preclinical results in breast cancer and glioma models [84,85], LS301 has entered phase I clinical trials (NCT04105062, NCT02807597) to evaluate its intraoperative safety and optimal dosing.

PLSWT7-DMI is another peptide-based fluorescent probe constructed by conjugating the bladder cancer-specific peptide PLSWT7 with the NIR dye IRDye800CW. Owing to the high affinity of the PLSWT7 peptide for bladder cancer-associated surface markers, the probe selectively accumulates in tumor tissues [86]. In a first-in-human phase I feasibility study for non-muscle-invasive bladder cancer (NMIBC), the probe demonstrated favorable safety without probe-related adverse events, supporting its feasibility for intraoperative imaging [87].

#### 3.1.3. Affibody-Based Probe in Phase I Clinical Trial

Beyond conventional antibodies and peptide ligands, engineered antibody mimetics such as affibodies have emerged as promising targeting platforms due to their smaller molecular size and improved tumor penetration.

ABY-029 is an EGFR-targeted NIR fluorescent probe composed of the affibody molecule Z03115-Cys conjugated to IRDye800CW, with excitation and emission maxima at 774 nm and 789 nm, respectively [88,89]. Compared with full-length antibodies, the smaller affibody scaffold provides favorable tissue penetration and rapid pharmacokinetics. It has previously been evaluated in phase 0 clinical trials in primary sarcoma, recurrent glioma, and head and neck tumors (NCT02901925, NCT03154411, NCT03282461), demonstrating feasibility for FGS at microdose levels. Samkoe et al. conducted a first-in-human study of the EGFR-targeted fluorescent Affibody probe ABY-029 in soft-tissue sarcoma patients. The study demonstrated that ABY-029 fluorescence intensity was positively correlated with EGFR expression levels, indicating that target abundance directly influenced intraoperative fluorescence signals [89]. Currently, a phase I clinical trial (NCT07279116) is ongoing in patients with head and neck squamous cell carcinoma to assess its safety, optimal dosing, and imaging performance.

#### 3.1.4. Functional and Multimodal Imaging Probe in Phase I Clinical Trial

Chen et al. developed a novel PET/NIR dual-modality imaging probe, ^68^Ga-IRDye800CW-BBN, which integrates the positron-emitting radionuclide ^68^Ga with the NIR dye IRDye800CW, thereby enhancing visualization of lower-grade gliomas [90]. A phase I clinical trial (NCT03407781) demonstrated that this probe enables effective integration of preoperative PET imaging with fluorescence-guided surgery (FGS) [91].

### 3.2. Probes in Phase II Clinical Trial

Phase II clinical trials focus on evaluating probe performance in specific tumor types, aiming to establish clinically effective strategies and to provide a solid foundation for subsequent large-scale clinical studies. The molecular structures, excitation wavelengths, and emission wavelengths of representative probes in phase II trials are summarized in Figure 4.

#### 3.2.1. Antibody-Based Targeted Probe in Phase II Clinical Trial

Among phase II clinical candidates, antibody-based fluorescent probes remain an important class due to their high molecular specificity and clinical translation potential. These probes mainly target tumor-associated receptors that are overexpressed on cancer cells.

Cetuximab-800CW is an EGFR-targeted fluorescent probe constructed by conjugating cetuximab with IRDye800CW, enabling precise labeling of EGFR-overexpressing lesions. A phase II clinical trial (NCT03134846) in oral squamous cell carcinoma demonstrated that Cetuximab-800CW enables highly sensitive detection of positive surgical margins, supporting its accuracy in real-time intraoperative margin assessment [92].

#### 3.2.2. Peptide-Based Targeted Probes in Phase II Clinical Trial

Compared with full-length antibodies, peptide-based probes offer smaller molecular size, favorable tissue penetration, and rapid pharmacokinetics.

cRGD-ZW800-1 is a targeted NIR peptide probe formed by conjugating a cyclic pentapeptide (cRGD) with the fluorophore ZW800-1. The cRGD motif specifically binds to integrin receptors that are widely expressed in tumor cells [93]. A study performed by de Valk et al. demonstrated that cRGD-ZW800-1 is safe and enables fluorescence imaging of colorectal cancer in both open and minimally invasive surgeries [93]. In a phase II clinical trial (NCT05518071), the probe was applied for intraoperative imaging of pancreatic and extrahepatic biliary tumors, with full clinical results not yet formally published.

EMI-137 is an NIR fluorescent probe consisting of a Cy5 fluorophore covalently linked to a peptide targeting the hepatocyte growth factor receptor (c-MET), with peak excitation and emission wavelengths of 653 nm and 675 nm, respectively. Its mechanism relies on specific binding of the peptide moiety to c-MET, which is overexpressed in various solid tumors, enabling tumor-specific accumulation [94,95]. In a phase IIa clinical trial (NCT03360461), EMI-137 demonstrated favorable safety. However, its relatively high background signal limited its clinical application [95].

#### 3.2.3. Small Molecule-Based Targeted Probes in Phase II Clinical Trial

In addition to antibody and peptide approaches, small molecule-based probes have attracted increasing attention due to their ability to target intracellular enzymes or tumor-associated proteins with high specificity.

PARPi-FL is a PARP1-targeted fluorescent imaging probe constructed by conjugating a PARP1 inhibitor scaffold with a BODIPY fluorophore. Due to overexpression of PARP1 in many tumor types, the probe selectively accumulates in tumor tissues, enabling high-contrast fluorescence imaging [96]. A phase I/II clinical study (NCT03085147) demonstrated favorable safety, along with enhanced tumor-to-normal tissue contrast. At the highest dose level (1000 nM), the probe achieved the greatest fluorescence enhancement, with tumor-to-margin fluorescence ratios exceeding 3 [97].

IS-002 is a prostate-specific membrane antigen (PSMA)-targeted NIR fluorescent probe currently in phase II clinical development. It is constructed by conjugating a PSMA ligand with an NIR fluorophore, with excitation and emission maxima at 774 nm and 793 nm, respectively [98,99]. Through selective binding to PSMA-expressing prostate cancer cells, IS-002 enables fluorescence visualization of primary lesions and associated lymph nodes. A phase I clinical trial (NCT04574401) demonstrated good tolerability and clear visualization of prostate cancer lesions and associated lymph nodes, while a multicenter, randomized phase II trial (NCT05946603) is currently evaluating its imaging performance in real surgical settings.

#### 3.2.4. Activatable Probes in Phase II Clinical Trial

Activatable fluorescent probes in phase II clinical development include protease-responsive and pH-responsive systems.

AVB-620 is an intravenously administered, ratiometric activatable fluorescent probe designed for the tumor microenvironment. It incorporates a fluorescence resonance energy transfer (FRET) system composed of Cy5 (far-red) and Cy7 (near-infrared) fluorophores linked via a peptide backbone. In its intact form, Cy5 fluorescence is quenched by Cy7; upon proteolytic cleavage by matrix metalloproteinases (MMPs) in the tumor microenvironment, the FRET effect is disrupted, resulting in enhanced Cy5 fluorescence and decreased Cy7 signal [100]. A phase I study demonstrated its safety and ability to detect tumor-positive tissues in humans [101]. A completed phase II clinical trial (NCT03113825) further showed that AVB-620 enables real-time monitoring of tumor tissues with high accuracy in distinguishing positive surgical margins [102].

ONM-100 is a nanoprobe based on polymeric micelles, composed of polyethylene glycol (PEG) and a polymethacrylate (PMMA) derivative covalently linked to indocyanine green (ICG) [103]. Under physiological pH conditions, the probe remains in a micellar state, with fluorescence quenched due to the covalent linkage of ICG to the polymer. Upon exposure to the acidic tumor microenvironment, the micelles dissociate ICG is released, and fluorescence is restored [103]. A multicenter phase II clinical trial in lung cancer demonstrated favorable safety. This study indicated the sensitivity and specificity of intraoperative molecular imaging (IMI) for detecting malignant tissue were 31.6% and 33.3%, respectively [104], indicating limited diagnostic performance in lung cancer applications. In another multicenter phase II study (NCT04950166), residual lesions were detected in 50% of patients (20/40), with no serious adverse events reported [105].

### 3.3. Probes in Phase III Clinical Trial

While phase II studies establish tumor-specific imaging performance, phase III trials are designed to validate clinical benefit in large patient populations and support regulatory approval. The molecular structures, excitation wavelengths, and emission wavelengths of representative probes are summarized in Figure 5.

#### 3.3.1. Antibody-Based Targeted Probe in Phase III Clinical Trial

SGM-101, an antibody–fluorophore conjugate targeting the carcinoembryonic antigen (CEA), is currently under phase III clinical evaluation. This probe consists of an anti-CEA monoclonal antibody labeled with a fluorophore, enabling selective accumulation in CEA-positive tumor tissues [106]. Vahrmeijer A.L. et al. investigated the application of SGM-101 for intraoperative imaging in pancreatic cancer [107]. In this study, 12 patients received preoperative intravenous infusion of SGM-101, followed by tumor visualization. The results showed a favorable safety profile, with only one patient experiencing mild discomfort. Moreover, SGM-101 also selectively accumulated in primary pancreatic tumors as well as hepatic and peritoneal metastases, with fluorescence signals correlating with regions of CEA expression [107].

#### 3.3.2. Peptide-Based Targeted Probe in Phase III Clinical Trial

BLZ-100 (generic name: tozuleristide) is a peptide-based targeted NIR probe developed using chlorotoxin as the targeting ligand. It exploits the tumor-targeting properties of chlorotoxin, which binds to matrix metalloproteinase-2 (MMP-2) and membrane-associated Annexin A2. By covalently conjugating chlorotoxin to an NIR fluorophore (ICG), BLZ-100 rapidly accumulates in malignant tissues such as gliomas following intravenous administration, generating high-contrast real-time fluorescence signals [108]. In a prior phase I clinical study (NCT02496065), BLZ-100 demonstrated good safety and feasibility for intraoperative visualization and margin assessment in breast cancer, with the potential to reduce reoperation rates [109]. Its ongoing phase II/III clinical trial (NCT03579602) focuses on pediatric central nervous system tumors to further validate its clinical efficacy in this population.

#### 3.3.3. Functional and Multimodal Imaging Probes in Phase III Clinical Trial

ASP5354 is a near-infrared imaging probe developed based on ICG analogs and designed for functional visualization of the urinary tract. Unlike tumor-targeted probes, ASP5354 relies on its pharmacokinetic properties, including rapid renal filtration and urinary excretion, to enable passive fluorescence visualization of ureters during surgery. A first-in-human phase I study (NCT03698305) evaluated its safety and pharmacokinetic properties, demonstrating a favorable safety profile, with fewer adverse events compared to placebo. Pharmacokinetic analysis showed that ASP5354 is rapidly filtered by the kidneys, accumulates in the urinary tract, and is excreted via urine, enabling passive visualization of the ureters [110]. A phase II study (NCT04238481) further established a dose–response relationship for imaging performance, providing clinical guidance for further development [111]. Currently, ASP5354 is being evaluated in phase III clinical trials (NCT05754333, NCT05999747) in patients undergoing abdominopelvic surgery.

Beyond visualization of internal organ structures, fluorescence-guided surgery has also been extended to the identification of functionally critical tissues, such as peripheral nerves, to minimize surgical injury. ALM-488 is a peptide-based fluorescent probe which consists of a nerve-targeting peptide conjugated with a fluorescent dye, enabling affinity for nerve tissues. A phase I/II clinical study (NCT04420689) evaluated its safety, pharmacokinetics, optimal dosing, and intraoperative imaging performance. The results showed a favorable safety profile with no dose-limiting toxicities observed across all dose levels. Pharmacokinetic analysis revealed a half-life of 29–72 min, with 500 mg identified as the optimal imaging dose, achieving the best signal-to-noise ratio while maintaining safety. In terms of imaging performance, ALM-488 enabled real-time fluorescence visualization of sensory and motor nerves, as well as their branches, with signal-to-background ratios (SBRs) significantly higher than those obtained with conventional white-light imaging [112]. The probe is currently undergoing phase III clinical evaluation (NCT05377554) in patients undergoing head and neck surgery to further assess its clinical utility.

The clinical development stages of these investigational fluorescent probes are summarized in Table 2.

## 4. Conclusions

FGS has rapidly evolved as an important intraoperative imaging strategy that provides real-time visualization beyond conventional white-light inspection. Clinically approved fluorescent probes, including indocyanine green (ICG), sodium fluorescein, methylene blue, 5-aminolevulinic acid (5-ALA), pafolacianine (OTL38), Akalux, and pegulicianine (LUM015), have demonstrated the feasibility and clinical value of fluorescence imaging across diverse surgical applications. These probes have contributed to improved visualization of anatomical structures, tumor localization, lymph node mapping, assessment of surgical margins, and identification of critical tissues, thereby providing surgeons with additional molecular or functional information during procedures. The successful clinical translation of these agents has established fluorescence imaging as a valuable adjunctive technology for enhancing surgical precision and safety.

Despite these achievements, currently approved fluorescent probes still face several limitations, including insufficient tumor specificity, limited tissue penetration, and nonspecific background signals. To overcome these challenges, fluorescent probes under clinical investigation are continuously evolving toward more sophisticated imaging strategies. Targeted probes have been developed to improve tumor selectivity through recognition of tumor-associated receptors, antigens, or enzymes, thereby enhancing tumor-to-background contrast. Meanwhile, activatable fluorescent probes further improve imaging specificity by generating fluorescence signals only after responding to tumor-specific stimuli, such as protease activity or acidic tumor microenvironments. In addition, functional imaging probes have expanded the role of fluorescence guidance beyond tumor detection by enabling visualization of critical anatomical structures, such as nerves and urinary tract structures, with the aim of improving surgical safety. These advances reflect a transition of FGS from nonspecific visualization toward biologically guided and precision-oriented surgical imaging.

Future development of fluorescent probes will focus on further improving imaging performance, integrating complementary technologies, and enabling intelligent surgical guidance. A primary structural focus is the development of fluorophores with longer emission wavelengths. Specifically, shifting optical imaging into the near-infrared II (NIR-II) window dramatically reduces photon scattering and tissue autofluorescence [113]. This facilitates deeper tissue penetration and superior imaging contrast, which are essential for visualizing deep-seated lesions and microscopic residual diseases. Recent advances in NIR-II fluorophores, including small-molecule organic dyes, and semiconductor quantum dots have demonstrated substantial improvements in brightness, photostability, and imaging depth. For example, alloyed quantum dots have achieved high photoluminescence quantum yields in the NIR-II window [114]. Unlike conventional core–shell passivation or heteroatomic doping strategies, which offer only limited or trade-off improvements, this alloying approach directly suppresses the intrinsic lattice defects responsible for poor fluorescence efficiency in Ag-based quantum dots, thereby achieving a record-high quantum yield together with a prolonged excited-state lifetime. Although inorganic and nanomaterial-based probes can provide high brightness and more prolonged photostability than small-molecule fluorophores, their more complex compositions, potential long-term retention, and uncertain metabolism may introduce additional challenges for toxicological evaluation and regulatory approval. Therefore, the most clinically promising NIR-II probes are likely to be those that achieve a balance between superior optical performance and well-characterized biological safety and pharmacokinetic profiles. Also, the clinical translation of NIR-II fluorescence imaging is not limited by the properties of fluorescent probes themselves. At the instrumentation level, NIR-II imaging requires sensitive detectors, appropriate excitation sources, optical filters, and dedicated imaging systems optimized for the longer-wavelength emission range. Thus, the future clinical translation of NIR-II fluorescence imaging will depend on coordinated advances in probe design and imaging instrumentation, rather than improvements in fluorophore performance alone.

Beyond advances in fluorescent probe design, multimodal imaging probes represent an emerging strategy to combine fluorescence imaging with other imaging modalities. For example, the combination of fluorescence and photoacoustic imaging provides enhanced anatomical and functional information, helping to address the limited tissue penetration of conventional optical imaging [115]. Furthermore, a redox-activatable MRI/NIR-II fluorescent probe was developed to integrate the high-resolution anatomical information of MRI with the real-time imaging capability of NIR-II fluorescence [116], which enabled preoperative localization of microlesions by MRI and intraoperative tumor margin delineation by NIR-II fluorescence imaging.

In parallel with the development of multimodal imaging platforms, artificial intelligence (AI)-assisted image analysis represents another promising direction for fluorescence-guided surgery. In current clinical practice, the interpretation of fluorescence signals still largely depends on surgeons’ experience, with a lack of standardized and quantitative evaluation frameworks. AI-based computational approaches, including deep learning-assisted image enhancement and automated signal analysis, can facilitate more objective fluorescence interpretation, improve tumor boundary delineation, and facilitate standardized surgical decision-making [117,118]. A representative MRI/NIR-II multimodal imaging strategy with AI-assisted analysis is shown in Figure 6.

## Figures and Tables

**Figure 1:**
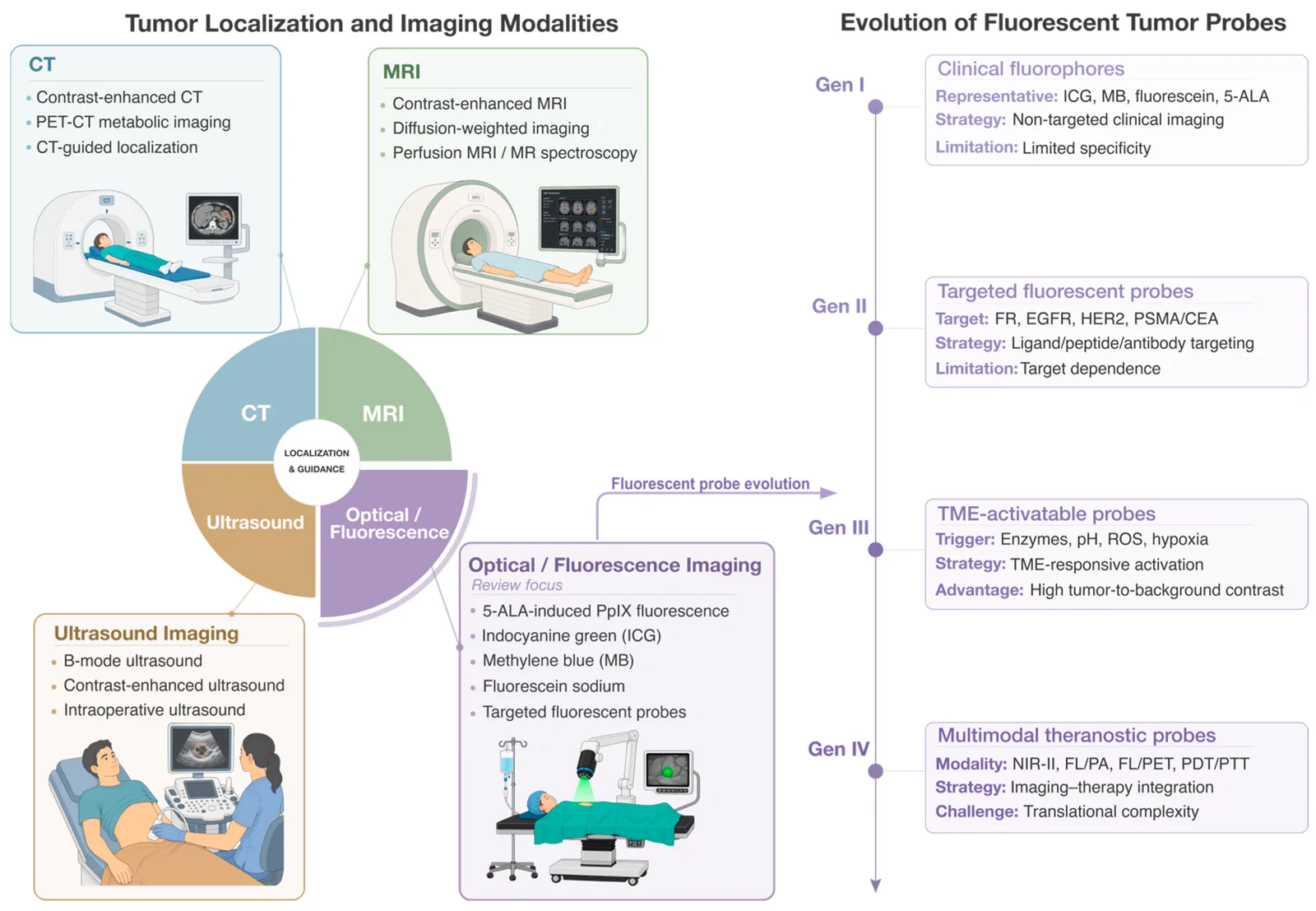
Imaging modalities for tumor localization and the development of fluorescent probes.

**Figure 2:**
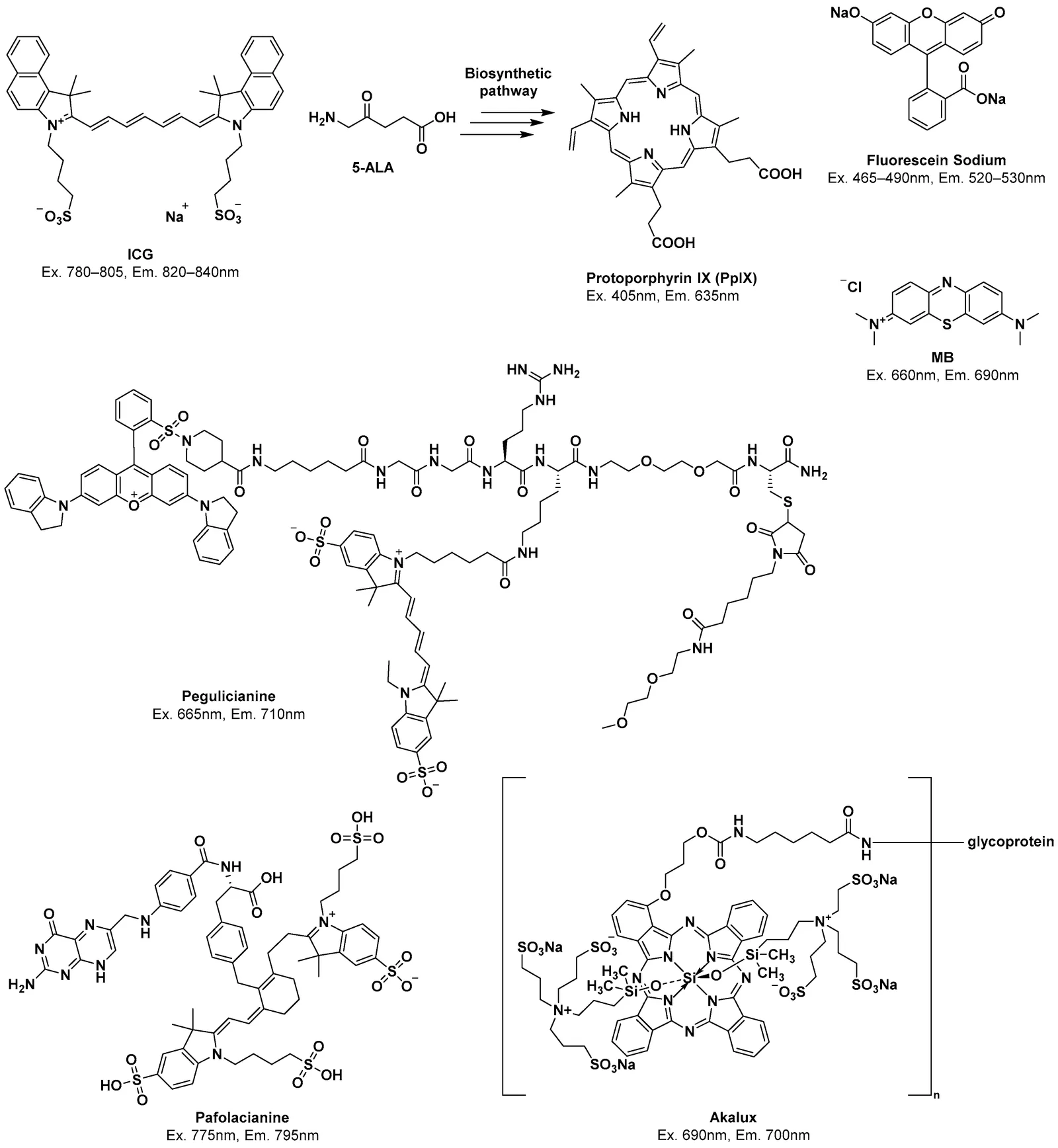
Molecular probes approved by FDA.

**Figure 3:**
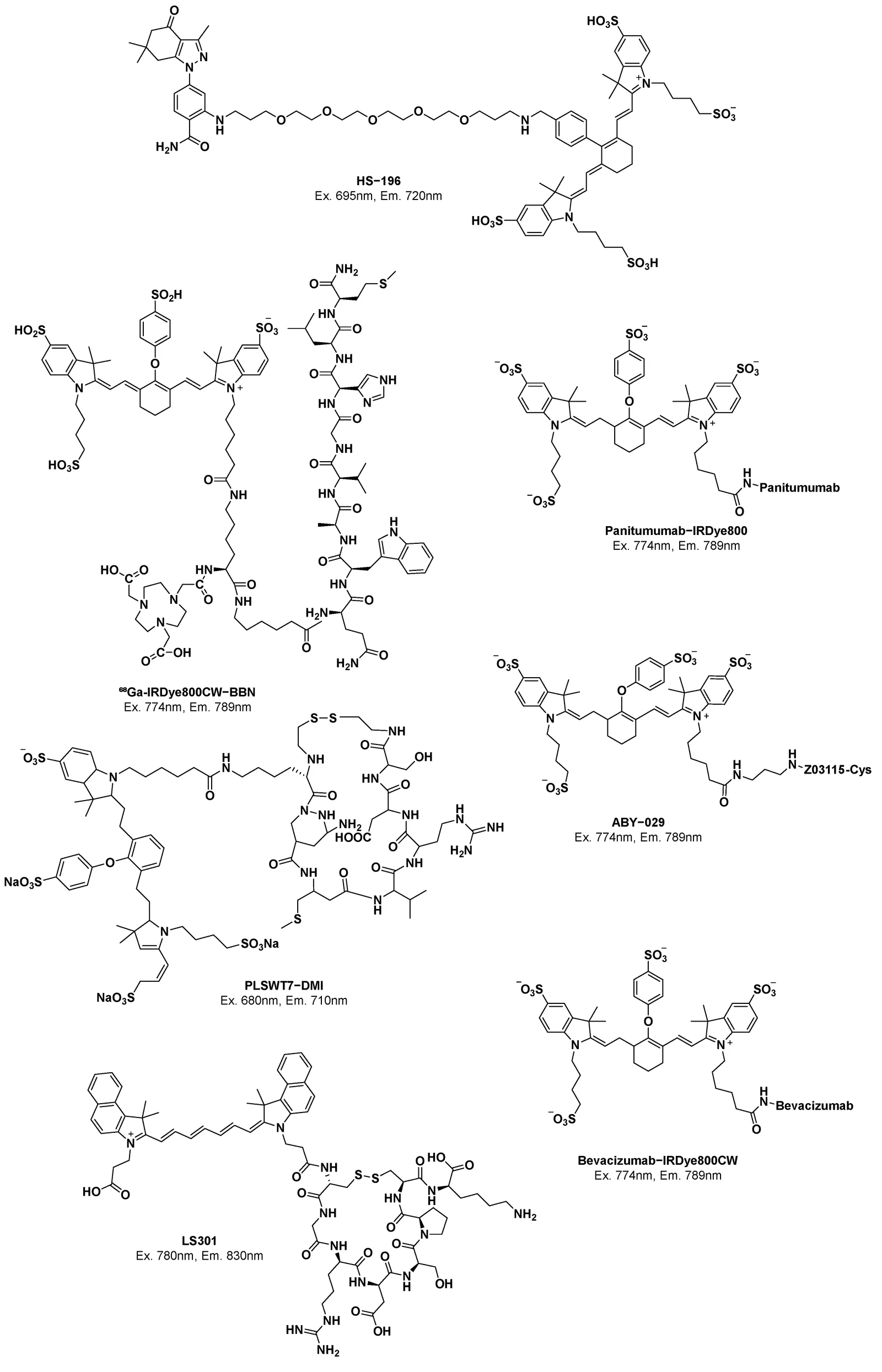
Probes in phase I clinical trial.

**Figure 4:**
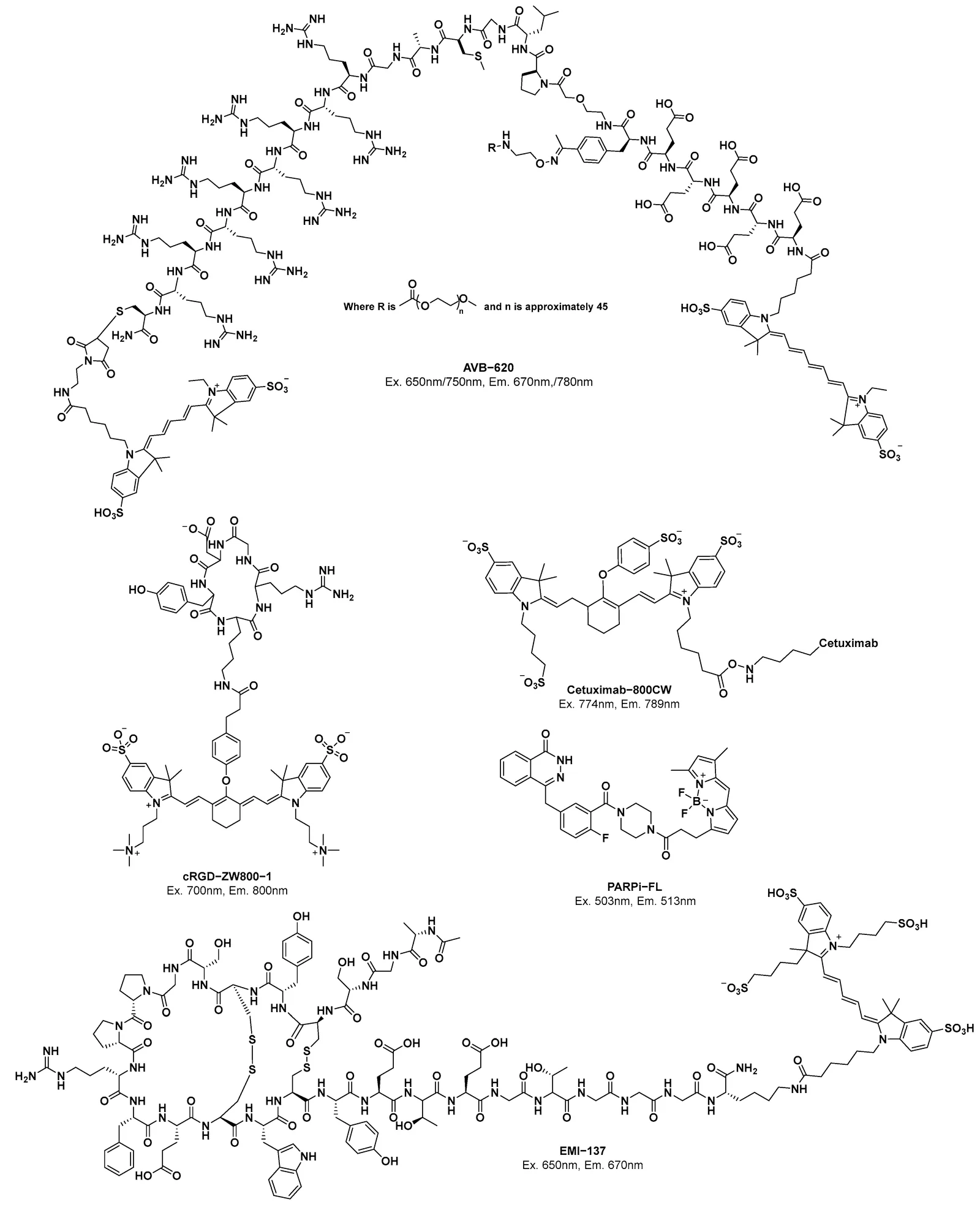
Probes in phase II clinical trial.

**Figure 5:**
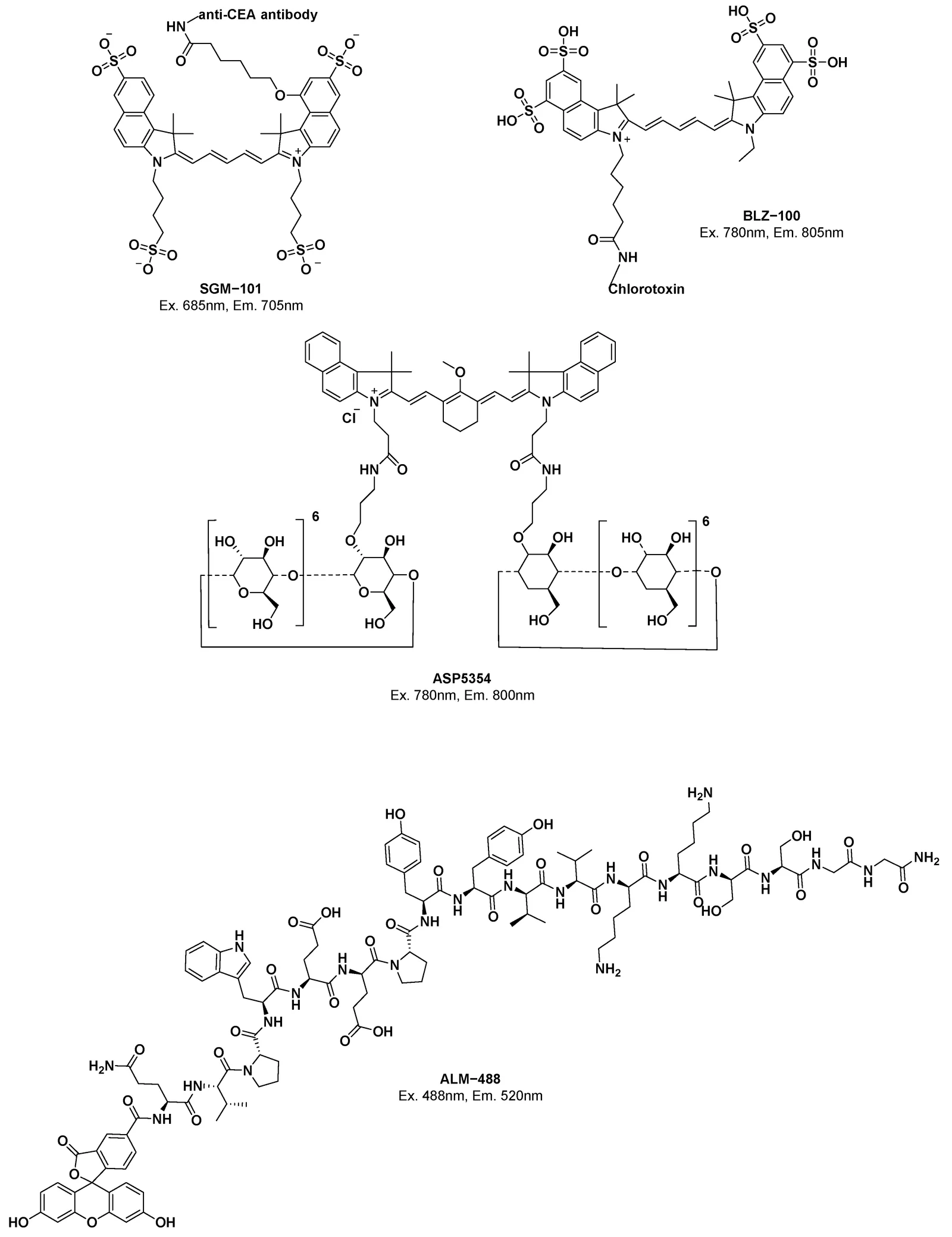
Probes in phase III clinical trial.

**Figure 6:**
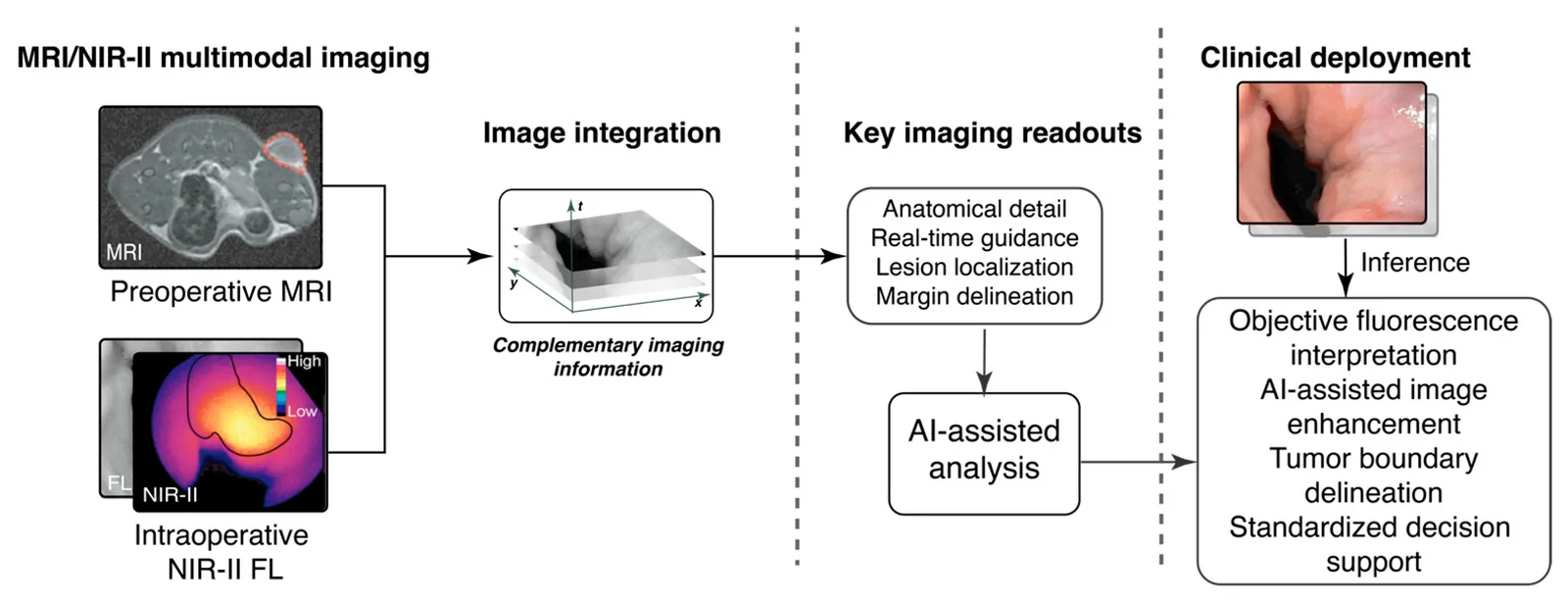
Representative MRI/NIR-II multimodal imaging and AI-assisted fluorescence-guided surgery. MRI and NIR-II fluorescence images reproduced from ref. [119], and the endoscopic image reproduced from ref. [120], both licensed under CC BY 4.0.

**Table 1:** Application of clinically used probes.

Probe Name	Applications
ICG	Laparoscopic common bile duct exploration [18]; anterior mediastinal tumors [19]; early-stage cervical cancer [20]; lung cancer [21]; colorectal liver metastases [22]
Sodium Fluorescein	Ophthalmology [30]; benign neuroepithelial brain tumors [31]; central nervous system tumors [32,33,34]; CSF leaks [35,36]
Methylene Blue	Breast cancer [41]; cervical cancer [42]; parathyroid adenoma [43,44]; small intestinal neuroendocrine tumors [45]
Pafolacianine	Ovarian cancer [49]; renal cell carcinoma [52]; gastric adenocarcinoma [53]; pituitary adenoma [54]; endometrial cancer [55]
Akalux	rHNSCC [63]; non-small cell lung cancer [64]
5-ALA	Gliomas [66]; non-muscle-invasive bladder cancer [70]; HAL [71]
Pegulicianine	Breast cancer [75]

**Table 2:** Probes in clinical trials and their applications.

Probe Name	Fluorophore	Applicable Tumor Types	Clinical Phase
Panitumumab-IRDye800	IRDye800CW	Glioma [77], breast cancer [77], head and neck squamous cell carcinoma (HNSCC) [78]	Phase I
Bevacizumab-IRDye800CW	IRDye800CW	Pancreatic cancer [79], breast cancer [80]	Phase I
HS 196	Cy7	Breast cancer (NCT03333031)	Phase I
LS301	cypate	Breast cancer [84], glioma [85]	Phase I
PLSWT7-DMI	IRDye800CW	Non-muscle-invasive bladder cancer (NMIBC) [87]	Phase I
ABY-029	IRDye800CW	Primary sarcoma (NCT02901925), recurrent glioma (NCT03154411), head and neck tumors (NCT03282461)	Phase I
^68^Ga-IRDye800CW-BBN	IRDye800CW	Glioma [90]	Phase I
Cetuximab-800CW	IRDye800CW	Oral squamous cell carcinoma (OSCC) [92]	Phase II
cRGD-ZW800-1	ZW800-1	Colorectal cancer [93], pancreatic cancer (NCT05518071), cholangiocarcinoma (NCT05518071)	Phase II
EMI 137	Cy5	Colorectal cancer (NCT03360461)	Phase IIa
PARPi-FL	BODIPY	Oral cancer (NCT03085147)	Phase II
IS-002	Not disclosed	Prostate cancer (NCT04574401, NCT05946603))	Phase II
AVB-620	Cy5/Cy7	Breast cancer (NCT03113825)	Phase II
ONM-100	ICG	Lung cancer [104,105]	Phase II
SGM-101	BM104	Colorectal cancer [106], pancreatic cancer [107]	Phase III
BLZ-100	ICG	Glioma [108], breast cancer [109], pediatric central nervous system tumors (NCT03579602)	Phase III
ASP5354	ICG analog	Ureter [110]	Phase III
ALM-488	Fluorescein-based	Head and neck squamous cell carcinoma (HNSCC) (NCT05377554)	Phase III

## Data Availability

No new data were generated or analysed for this review. All information discussed is based on previously published literature cited in the article.
